# Study on the Storage Stability and Rheological Property of Bio-Oil/Lignin Composite-Modified Asphalt

**DOI:** 10.3390/polym16172484

**Published:** 2024-08-30

**Authors:** Guixiu Guo, Junfeng Gao, Dongzhao Jin, Xuan Wang, Yanqiu Bi, Peng Guo

**Affiliations:** 1 National & Local Joint Engineering Research Center of Transportation and Civil Engineering Materials, Chongqing Jiaotong University, Chongqing 400074, China; gxguo@mails.cqjtu.edu.cn (G.G.); biyanqiu@cqjtu.edu.cn (Y.B.); 2 Civil, Environmental, and Geospatial Engineering, Michigan Technological University, 1400 Townsend Drive, Houghton, MI 49931-1295, USA; 3 School of Materials Science and Engineering, Chongqing Jiaotong University, Chongqing 400074, China; wangxuan02@mails.cqjtu.edu.cn (X.W.); guopeng@cqjtu.edu.cn (P.G.)

**Keywords:** bio-oil, lignin, modified asphalt, storage stability, rheological property

## Abstract

The objective of this study is to investigate the storage stability and rheological property of bio-oil/lignin composite-modified asphalt. The composite-modified asphalt with different proportions of bio-oil was prepared and cured at 105 °C, 135 °C, and 165 °C for 24 h and 48 h. The storage stability of the composite-modified asphalt was evaluated based on the softening point difference, the storage stability index derived from rotational viscosity, the segregation rate based on temperature sweep, and the non-recoverable creep compliance measured through the Multiple Stress Creep Recovery test. The storage stability of bio-oil/lignin composite-modified asphalt was evaluated through testing and analysis of its infrared spectroscopy and scanning electron microscopy before and after thermal storage. The research results indicate that the maximum difference in softening point is 0.9 °C, and the calculated storage stability index is generally below 0.1. The maximum value of the segregation rate is 0.43, indicating excellent storage stability of the bio-oil/lignin composite-modified asphalt. According to the results from infrared spectroscopy, no chemical reactions occurred during the storage process of the composite-modified asphalt. The scanning electron microscope confirmed that the samples became more stable after 48 h of storage.

## 1. Introduction

The total mileage of China’s expressways is about 177,000 km, and the total mileage of highways is nearly 5.4 million kilometers. In the field of road construction, the consumption of petroleum asphalt is approximately 33 million tons, and the price of petroleum asphalt has been increasing year by year [1,2]. As a result, an increasing number of researchers and scholars are exploring sustainable development in road construction and reducing dependence on petroleum resources [3,4,5]. One solution being explored is the use of crushed waste tires and rubber as a partial replacement for asphalt in road construction [6,7,8,9]. Additionally, the conversion of renewable biomass materials into bio-oil through high-temperature pyrolysis as a substitute for some of the asphalt is another approach under investigation [10,11,12]. Biomass materials have a wide range of sources, including agricultural crop residues [13], animal manure [14], waste cooking oil [15], and more. These materials are abundantly available, cost-effective, and have large reserves.

Bio-oil and lignin, as important components of biomass resources, possess excellent renewability, environmental friendliness, and degradability [16]. There have been several studies focused on the use of bio-oil or lignin for modifying asphalt, primarily concentrating on the changes in the performance of the base asphalt. In the early studies, Fini et al. [17] investigated the feasibility of using bio-oil derived from pig manure via the pyrolysis liquefaction process for road applications. The research indicated that bio-oil can enhance the low-temperature performance of petroleum asphalt and reduce road construction costs. You et al. [18] prepared bio-asphalt using wood pyrolysis oil and found that bio-oil can improve the fatigue performance of asphalt mixtures but may decrease the resistance to rutting at high temperatures. At low dosages (<10%), bio-oil can be used as a modifier for petroleum asphalt. Ding et al. [19] discovered that the performance of 50# petroleum asphalt with a 10% bio-oil content is comparable to that of 70# petroleum asphalt. Batista [20] conducted research on the physical and chemical properties of lignin-modified asphalt and found that lignin can significantly improve the high-temperature performance of asphalt. Gao et al. [21] reached the same conclusion in their study. Yu et al. [22] investigated the impact of lignin content on the performance of base asphalt and found that lignin-modified asphalt exhibits excellent resistance to rutting and fatigue at high temperatures. However, lignin can negatively affect the low-temperature performance of the base asphalt, and the higher the lignin content, the greater the damage. Zhang et al. [23] partially replaced petroleum asphalt with bio-oil and lignin and studied the performance of the modified asphalt. They found that the high-temperature and low-temperature performance of the modified asphalt were improved. This demonstrated the potential of bio-oil and lignin as additives for asphalt. Xue et al. [24] found that lignin increased the elastic component of asphalt, while waste engine oil reduced the elastic component of asphalt. Lignin and waste engine oil improved the high-temperature and low-temperature performance of asphalt at certain dosages. In the study by Fakhri et al. [25], lignin enhanced the resistance to permanent deformation of asphalt but had a negative impact on its low-temperature performance. The addition of waste engine oil resulted in improved performance of the modified asphalt at both high and low temperatures. In existing research, bio-oil has been shown to improve the low-temperature performance of asphalt but has adverse effects on its high-temperature performance. On the other hand, lignin has the opposite effect. Lignin can enhance the aging resistance and improve the high-temperature performance of asphalt but has a negative impact on its low-temperature performance. Therefore, combining bio-oil and lignin for asphalt modification can help compensate for the respective drawbacks in their performance.

Modified asphalt is typically a composite material composed of a dispersed phase of modifiers and a continuous phase of base asphalt, forming a multiphase structural system [26]. Liang et al. [27] studied the phase separation between modifier and asphalt by phase field theory and divided it into three stages: development, aggregation, and gravity induction. During storage, the modified asphalt is prone to phase separation due to intermolecular forces, leading to the aggregation or precipitation of modifiers within the asphalt [28]. In the study by Wen et al. [29], the density difference between rubber modifiers and asphalt is also an important reason for segregation. This phenomenon can significantly impact the performance and stability of the modified asphalt. Therefore, studying the storage stability of modified asphalt is of paramount importance [30,31]. However, relying solely on the traditional evaluation indicator of softening point difference to assess the storage stability of bio-oil/lignin composite-modified asphalt is rarely simplistic. This study aims to investigate the storage stability and rheological property of bio-oil/lignin composite-modified asphalt. The evaluation indicators include softening point difference, storage stability index (Is), segregation rate (Rs), and irreversible creep compliance (Jnr). Techniques such as Fourier-transform infrared spectroscopy and scanning electron microscopy were also employed to investigate the storage stability of bio-oil/lignin composite-modified asphalt. This paper will provide insights for the future storage and application of bio-oil/lignin composite-modified asphalt.

## 2. Materials and Methods

### 2.1. Raw Materials and Preparation of Sample

#### 2.1.1. Materials

The base asphalt is 70# petroleum asphalt provided by China Petroleum & Chemical Corporation (Beijing, China) and the technical parameters are shown in Table 1. Bio-oil is derived from waste wood chips, which are liquid at room temperature and dark brown in color. Bio-oil has a similar elemental composition to asphalt and exhibits good compatibility with asphalt [32,33]. This compatibility facilitates the mixing and interaction between bio-oil and asphalt, thereby promoting the formation of composite-modified asphalt [34]. Lignin is provided by Tianjin Damao Reagent Factory (Tianjin, China) and is a white flocculent.

#### 2.1.2. Sample Preparation

Lignin and base asphalt were subjected to high-speed shear at 135 °C for 10 min, followed by the gradual addition of varying proportions of bio-oil. The shearing process was continued for an additional 30 min to ensure the homogeneous blending of lignin, bio-oil, and base asphalt. The lignin content is 0.3% of the mass of the base asphalt, while the bio-oil content is 1% and 2% of the mass of the base asphalt. The shearing rate is 3000 revolutions per minute.

### 2.2. Thermal Storage Stability Test

**Figure 1:**
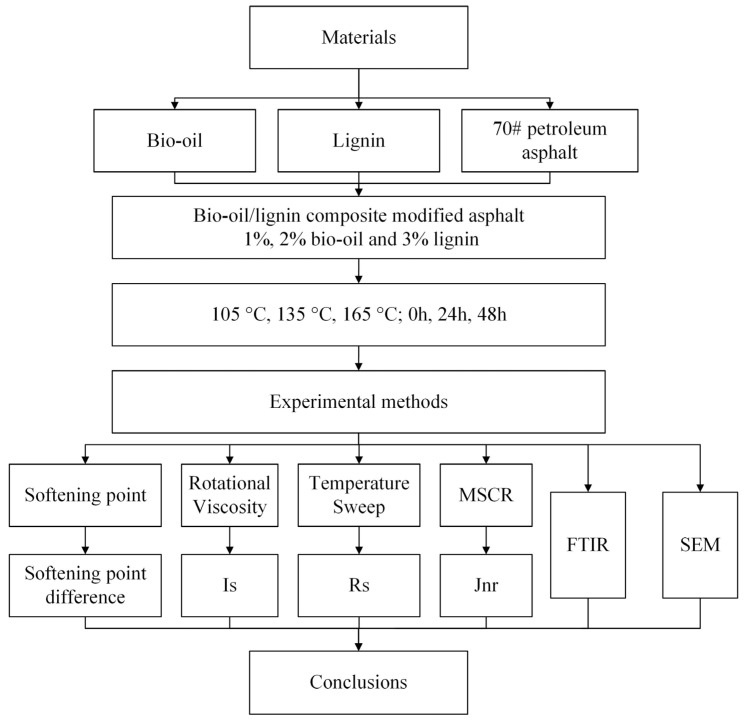
The flow chart of the research approach.

Two groups of bio-oil/lignin composite-modified asphalt with different proportions were poured into aluminum tubes with a diameter of 2.5 cm and a height of 14 cm. The aluminum tubes containing the asphalt samples were stored at different temperatures (105 °C, 135 °C, 165 °C) for various durations (0, 24 h, 48 h). After storage, the aluminum tubes were removed and cooled and then cut into three equal portions. Samples were taken from the top and bottom parts of the aluminum tubes containing the bio-oil/lignin composite-modified asphalt to evaluate their storage stability based on physical properties, rheological characteristics, and functional group composition. If there is minimal difference in performance between the top and bottom portions of the bio-oil/lignin composite-modified asphalt, it indicates good stability of the samples. Conversely, if there is a significant difference, it suggests inadequate storage stability of the composite-modified asphalt. The experimental flowchart of this study is shown in Figure 1.

### 2.3. Physical Properties Characterization

#### 2.3.1. Softening Point Test

The softening point test is conducted using the ring-and-ball method. By testing the softening points of samples from the top and bottom of the aluminum tube, the absolute difference in softening points between the top and down parts, denoted as ΔTR&B, is obtained. This value can be used to evaluate the segregation of the bio-oil/lignin composite-modified asphalt. When the absolute difference in softening points (ΔTR&B) is less than 2.5 °C, the storage stability meets the standard requirements [36].

(1) $$\Delta TR\&B=\left|{TR\&B}_{t}\right.-\left.{TR\&B}_{b}\right|$$

where TR&B*_t_* is the softening point at the top of the specimen, °C; TR&B*_b_* is the softening point at the bottom of the specimen, °C.

#### 2.3.2. Rotational Viscosity Test

The viscosity of the bio-oil/lignin composite-modified asphalt samples in the top and bottom parts of the aluminum tube was tested by Brookfield viscometer at different temperatures (90 °C, 105 °C, 120 °C, 135 °C). The storage stability index (Is) is calculated according to Equation (2) to evaluate the storage stability of bio-oil/lignin composite-modified asphalt. The smaller the Is result, the better the storage stability of the asphalt [37].

(2) $$Is=(\mu_{t}-\mu_{b})/\mu_{0}$$

where *μ_t_* and *μ_b_* are the viscosity of the top and bottom composite-modified asphalt of the aluminum tube, respectively, and *μ*_0_ is the viscosity of the composite-modified asphalt that is not stored.

### 2.4. Rheological Property Evaluation

#### 2.4.1. Temperature Sweep Test

The incorporation of bio-oil will affect the high-temperature performance of the modified asphalt, and the temperature of the modified asphalt before and after thermal storage is evaluated by Dynamic Shear Rheometer. The curves of complex modulus (G*), phase angle (*δ*), and rutting factor (G*/*sinδ*) with temperature are established to explore the high-temperature rheological property and temperature variation of bio-oil/lignin composite-modified asphalt. The test temperature range is selected as 52 °C~82 °C, and the interval is 6 °C. A 1% strain level is selected with a loading frequency of 10 rad/s. According to the test results, the storage rate Rs of asphalt was calculated to evaluate the heat storage stability of asphalt. The closer the Rs is to 0, the better the storage stability of the asphalt [38].

(3) $${Rs=(G*/sin\delta )}_{b}/{\left(G*/sin\delta \right)}_{t}-1$$

where (G*/*sinδ*)*_t_* and (G*/*sinδ*)*_b_* are the rutting factors at the top and bottom of the aluminum tube.

#### 2.4.2. Multiple Stress Creep Recovery (MSCR) Test

The multiple stress creep mode of Dynamic Shear Rheometer is selected, and the non-recoverable creep compliance (*Jnr*) is used as the technical index to evaluate the high-temperature performance of composite-modified asphalt. Under the two stress levels of 0.1 kPa and 3.2 kPa, the top and down asphalt samples are tested for 10 cycles; each cycle is 10 s, which is divided into 1 s loading creep stage and 9 s unloading recovery stage for a total loading of 200 s. The temperature selected for the test is 64 °C, the shear strain at 0.1 kPa and 3.2 kPa is obtained, the *Jnr* in the period is calculated by Equation (4), and its average value is calculated as the final result.

(4) $$J_{nr}=\frac{\varepsilon_{nr}}{\sigma }$$

where *ε_nr_* is the non-recoverable strain, and *σ* is the stress level, 0.1/3.2 kPa.

### 2.5. Fourier-Transform Infrared (FTIR) Spectroscopy

The functional groups of the samples at the top and bottom ends of the test tubes were analyzed using the NICOLETiS20 infrared spectrometer, a brand by Thermo Fisher Scientific (Waltham, MA, USA). The detector is DLaTGS, the resolution is 4 cm^−1^, the spectral scanning range is 4000~400 cm^−1^, and the number of scans is 32 times.

### 2.6. Scanning Electron Microscopy (SEM)

The experiment utilized a Zeiss Sigma 300 model scanning electron microscope (SEM) with an accelerating voltage ranging from 0.02 to 30 kV. The equipment is from the ZEISS Group (Oberkochen, Germany). The magnifications chosen were 34–37× and 500×. Firstly, the composite-modified asphalt with a bio-oil content of 1% was scanned. Subsequently, the asphalt samples from the top and bottom of the aluminum tubes were scanned after 24 h and 48 h of storage at a temperature of 165 °C. This was performed to observe the morphology of the composite-modified asphalt.

## 3. Results

### 3.1. Softening Point Difference

**Figure 2:**
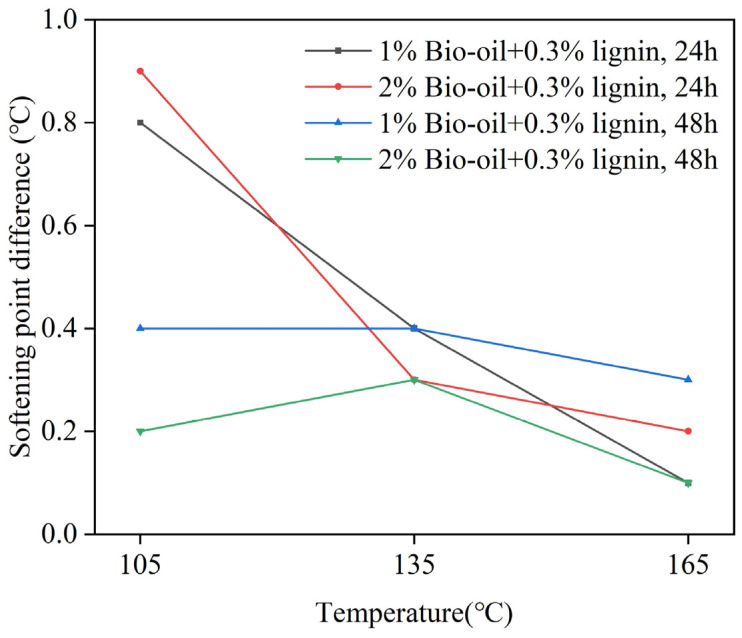
Softening point difference of bio-oil/lignin composite-modified asphalt.

The softening point reflects the high-temperature properties of the asphalt. In this study, the bio-oil/lignin composite-modified asphalt samples were subjected to softening point measurements at both the top and bottom sections of the aluminum tube. The difference in softening points was calculated to evaluate the homogeneity and storage stability of the modified asphalt. A smaller softening point difference indicates a closer performance between the top and bottom sections of the modified asphalt, indicating better storage stability [39]. The softening point of different asphalts after thermal storage at different temperatures are presented in Table 2., while Figure 2 illustrates the softening point difference of the bio-oil/lignin composite-modified asphalt.

The lower softening point observed in the composite-modified asphalt compared to the base asphalt can be attributed to the presence of a significant amount of lightweight components in the bio-oil. These lightweight components act as diluents for the asphalt, affecting its overall softening point [40]. Following the aluminum tube separation test, the softening point of the bio-oil/lignin composite-modified asphalt was found to be higher than the softening point prior to the separation test. This increase in softening point can be attributed to the possibility of oxidation occurring during the thermal aging process. This oxidation process can lead to the conversion of the lightweight components in the asphalt into heavier components, resulting in increased hardness and viscosity of the asphalt [32,41]. The softening point of the bottom portion of the stored asphalt samples typically exhibits higher values compared to the top portion. This phenomenon can be attributed to the precipitation and separation of heavier components during the thermal storage process. Figure 2 shows that when the bio-oil content is 2% and the samples undergo 24 h of thermal storage at 105 °C, the maximum softening point difference (ΔTR&B) is 0.9 °C, which falls below the threshold of 2.5 °C. These results indicate that the bio-oil/lignin composite-modified asphalt demonstrates favorable storage stability. Moreover, when subjected to a storage temperature of 165 °C, the softening point difference is minimal, measuring only 0.1 °C. These findings highlight the robust storage stability of the bio-oil/lignin composite-modified asphalt under different thermal conditions.

### 3.2. Rotational Viscosity and Is

**Figure 3:**
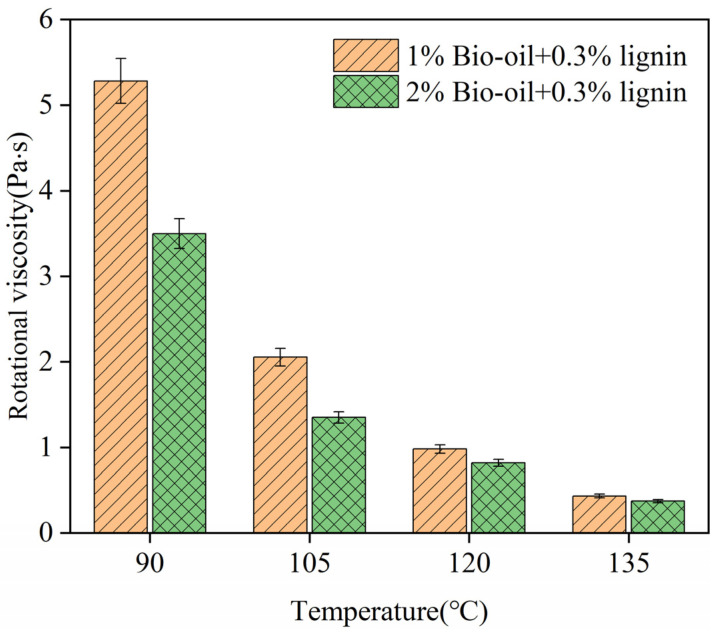
The rotational viscosity of bio-oil/lignin composite-modified asphalt.

**Figure 4:**
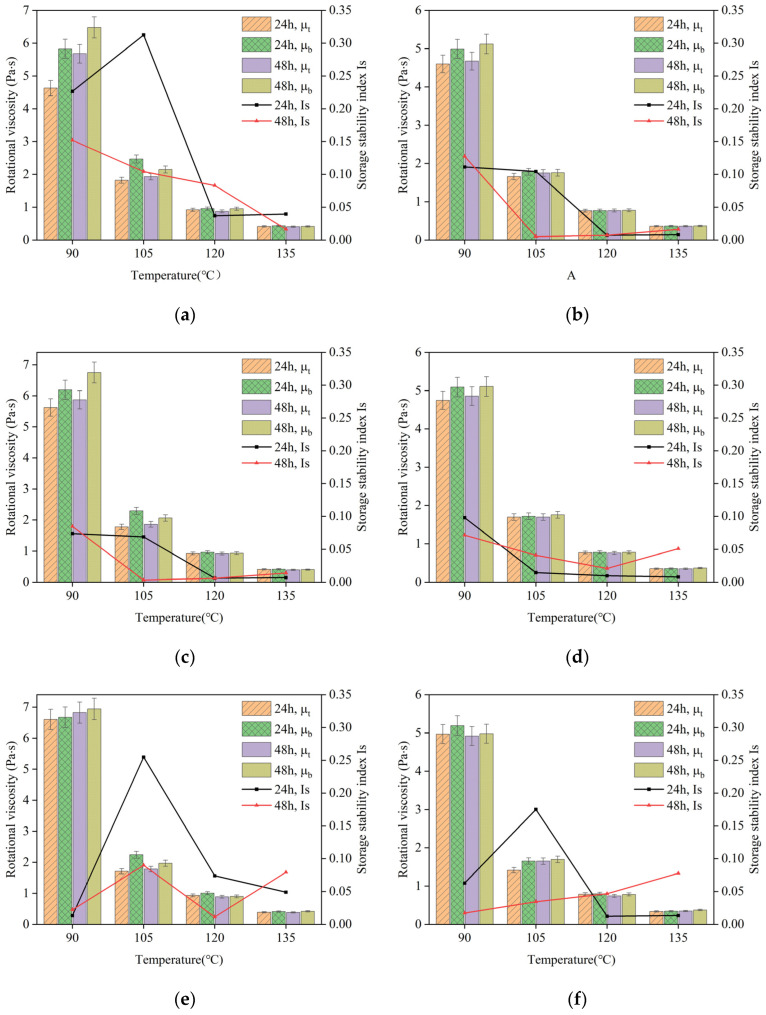
The rotational viscosity of composite-modified asphalt and the storage stability index (Is): (**a**) 105 °C, 1% bio-oil + 0.3% lignin; (**b**) 105 °C, 2% bio-oil + 0.3% lignin; (**c**) 135 °C, 1% bio-oil + 0.3% lignin; (**d**) 135 °C, 2% bio-oil + 0.3% lignin; (**e**) 165 °C, 1% bio-oil + 0.3% lignin; (**f**) 165 °C, 2% bio-oil + 0.3% lignin.

Viscosity is a measure of the internal molecular frictional resistance experienced by asphalt during flow, indicating its ability to resist deformation. In this study, the viscosity of the bio-oil/lignin composite-modified asphalt was evaluated before thermal storage (Figure 3) and after 24 h and 48 h of thermal storage. The storage stability index (Is) was calculated to assess the changes in viscosity over time (Figure 4).

In Figure 2, as the temperature increases, the viscosity of the asphalt decreases, indicating a decrease in shear resistance. Taking the composite-modified asphalt with 2% bio-oil content as an example, the viscosities at 90 °C, 105 °C, 135 °C, and 165 °C are 5.28 Pa·s, 2.05 Pa·s, 0.98 Pa·s, and 0.43 Pa·s, respectively. Moreover, an increase in bio-oil content leads to a decrease in viscosity for the modified asphalt at the same test temperature, with a noticeable distinction observed at 90 °C. Specifically, the viscosity of the composite-modified asphalt with a 2% bio-oil content is approximately two-thirds that of the composite-modified asphalt with a 1% bio-oil content. With the increase in temperature, the disparity in viscosity between the asphalt samples diminishes. At 135 °C, the impact of bio-oil content on asphalt viscosity becomes less significant. This is consistent with the conclusion of Xue et al. [24]. These observations highlight the temperature-dependent behavior of asphalt viscosity and the influence of bio-oil content on the viscosity of the composite-modified asphalt.

Based on the observations presented in Figure 4, which depicts the viscosity and Is of different dosages of composite-modified asphalt at storage temperatures of 105 °C, 135 °C, and 165 °C, it can be noted that the viscosity of the asphalt at the bottom of the aluminum tube is higher than that at the top. This finding aligns with the results obtained from the softening point difference test and suggests the possible migration of heavy asphalt components toward the bottom portion under high-temperature conditions. This phenomenon contributes to understanding the behavior of the composite-modified asphalt and provides insights into the distribution of components within the material during thermal storage.

In terms of the dosage of bio-oil in the composite-modified asphalt, it is observed that the majority of the asphalt samples with a bio-oil content of 2% exhibit lower Is values compared to those with a bio-oil content of 1%. The closer the Is value is to 0, the better the storage stability of the composite-modified asphalt [37]. This suggests that the composite-modified asphalt with a 2% bio-oil content demonstrates smaller changes in viscosity between the top and bottom sections of the aluminum tube after undergoing the thermal storage process, indicating better storage stability. Additionally, when considering the storage temperature, the Is at 135 °C generally exhibits lower values compared to those at 105 °C and 165 °C. These findings provide insights into the impact of bio-oil dosage and storage temperature on the storage stability of the composite-modified asphalt.

### 3.3. Temperature Sweep Test and Rs

**Figure 5:**
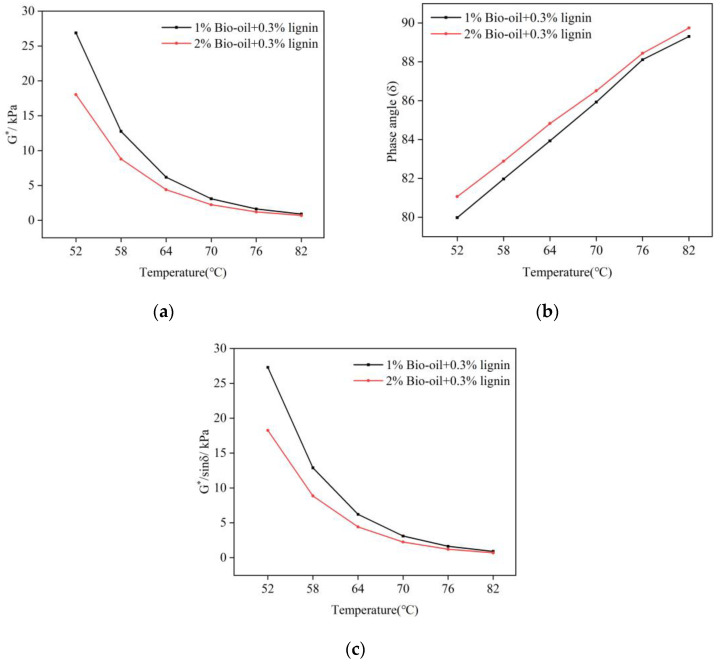
Temperature scan results of bio-oil/lignin composite-modified asphalt: (**a**) complex modulus (G*); (**b**) phase angle (*δ*); (**c**) rutting factor (G*/*sinδ*).

The temperature scan experiment employs complex modulus (G*) and phase angle (*δ*) as evaluation parameters to assess the rheological property of the modified asphalt. G* represents the modified asphalt’s ability to resist deformation under shear load, while δ indicates the relative magnitude of the elastic and viscous components of the modified asphalt. The rutting factor (G*/*sinδ*) signifies the modified asphalt’s resistance to deformation at high temperatures [42]. A higher value of G*/*sinδ* indicates superior resistance to high-temperature deformation. Figure 5 illustrates the variation of complex modulus, phase angle, and rutting factor of the composite-modified asphalt with the addition of 1% bio-oil and 2% bio-oil as a function of temperature.

As the temperature increases, the complex modulus of the composite-modified asphalt decreases, which is shown in Figure 5. Moreover, an increase in the added amount of bio-oil leads to a decrease in the complex modulus of the modified asphalt. This reduction suggests a decrease in the deformability resistance of the bio-oil-modified asphalt as the bio-oil content and temperature increase. The influence of bio-oil content on the complex modulus becomes less pronounced at higher temperatures. Simultaneously, as the bio-oil content increases, the phase angle of the asphalt also increases, indicating a decrease in its resistance to rutting deformation. Notably, modified asphalt with a bio-oil content of 2% exhibited a lower rutting factor. This is consistent with the results of Zhang et al. [23], indicating that the addition of bio-oil reduced the high-temperature performance of the modified asphalt.

**Figure 6:**
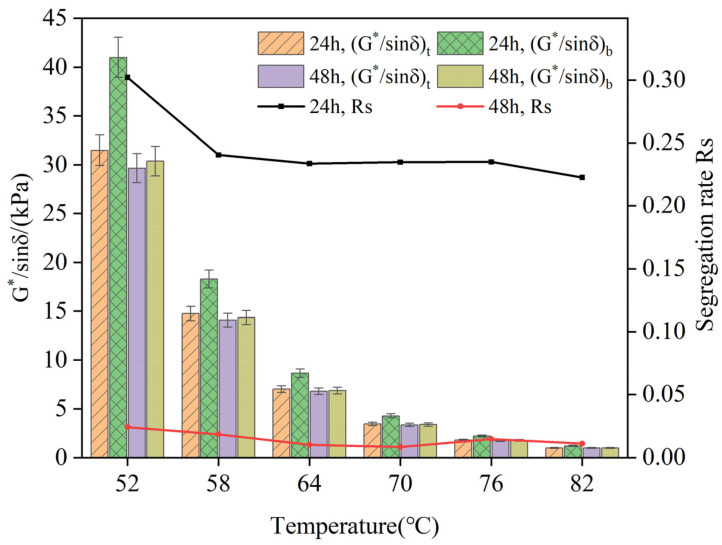
Rutting factor and Rs of composite-modified asphalt under different storage times.

Figure 6 presents the rutting factor and segregation rate (*Rs*) of the composite-modified asphalt with a 1% bio-oil content on the top and bottom sections of the aluminum tube after storage at 105 °C for 24 h and 48 h.

Figure 6 demonstrates that the rutting factor of the composite-modified asphalt in the bottom section of the aluminum tube is higher compared to that in the top section at the same storage temperature. This observation aligns with the results obtained from the softening point and viscosity tests, further confirming the consistency in the behavior of the composite-modified asphalt throughout the different evaluations. The migration of lightweight components in the composite-modified asphalt is expected during storage. The calculated segregation rate (*Rs*) after 24 h of storage ranged between 0.2 and 0.3, while for 48 h of storage, the Rs values ranged from 0 to 0.05. Interestingly, the calculated Rs for the 24 h storage period was higher than that for the 48 h storage period, indicating improved stability of the composite-modified asphalt after 48 h of storage. These observations highlight the dynamics of lightweight component migration and suggest that a longer storage duration enhances the stability of the composite-modified asphalt.

**Figure 7:**
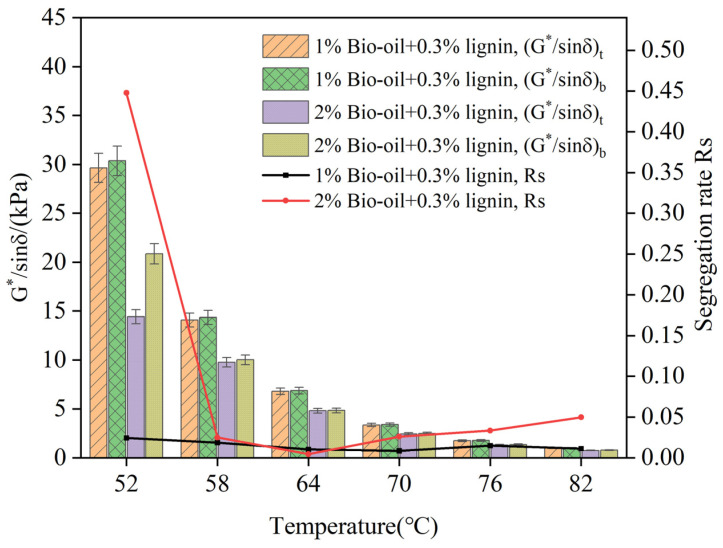
Rutting factor and Rs of composite-modified asphalt under different bio-oil contents.

In order to study the effect of bio-oil content on the storage stability of modified asphalt, the temperature sweep test of the composite-modified asphalt in the top and bottom parts of the aluminum tube after storage at 105 °C for 48 h was carried out, and the rutting factor and Rs were calculated, as shown in Figure 7.

The increase in bio-oil content leads to a decrease in the rutting factor of the composite-modified asphalt, and a significant decrease at lower test temperatures is shown in Figure 7. The test results of the *Rs* values between 58 °C and 82 °C, with varying dosages of bio-oil, exhibit relatively similar trends. The presence of bio-oil content has a minimal impact on the storage stability of the composite-modified asphalt. The Rs values obtained across the temperature range were consistently small, indicating good storage stability and a lack of significant segregation in the composite-modified asphalt. These findings suggest that the addition of bio-oil does not significantly affect the storage stability of the composite-modified asphalt within the tested temperature range.

**Figure 8:**
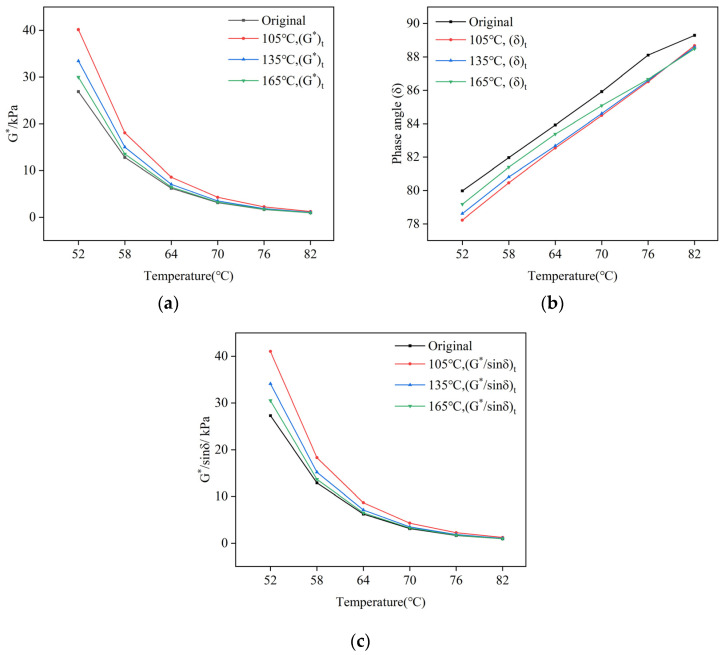
Temperature sweep results of composite-modified asphalt on the top part of aluminum tube at different storage temperatures: (**a**) complex modulus (G*); (**b**) phase angle (*δ*); (**c**) rutting factor (G*/*sinδ*).

The temperature sweep of the composite-modified asphalt of the top and bottom parts of the aluminum tube stored for 24 h at different storage temperatures is carried out, and the complex modulus, phase angle, and rutting factor are shown in Figure 8.

Based on the findings presented in Figure 7, it is observed that the complex modulus of the composite-modified asphalt increases after undergoing thermal storage. Simultaneously, the phase angle decreases while the rutting factor increases. Additionally, when considering different storage temperatures, the rutting factors of the composite-modified asphalt in the top section of the aluminum tube exhibit a descending order of 105 °C > 135 °C > 165 °C.

### 3.4. MSCR Test

**Figure 9:**
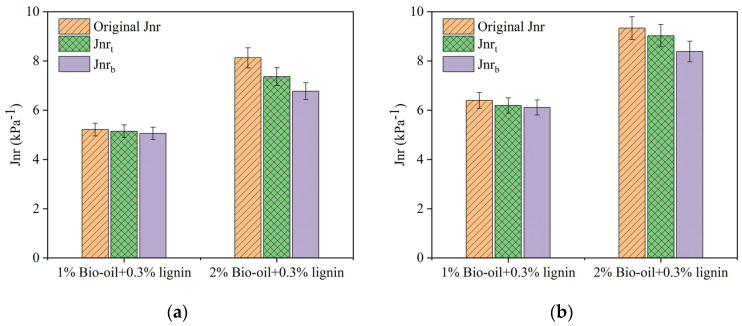
Jnr of composite-modified asphalt under different bio-oil contents: (**a**) 0.1 kPa; (**b**) 3.2 kPa.

Compared with the rutting factor G*/*sinδ*, the MSCR test can well characterize the rutting resistance of the modified asphalt pavement [43]. Figure 9 shows the non-recoverable creep compliance (*Jnr*) of different bio-oil dosages stored at 105 °C for 48 h.

Figure 9 reveals that the non-recoverable creep compliance (*Jnr*) of the bio-oil/lignin composite-modified asphalt in both the top and bottom sections of the aluminum tube is lower after undergoing thermal storage compared to the *Jnr* before storage. This reduction indicates an enhancement in the asphalt’s resistance to rutting following the thermal storage process. Furthermore, at the same stress level, the *Jnr* of the composite-modified asphalt with a 1% bio-oil content is lower than that of the asphalt with a 2% bio-oil content, implying that bio-oil has a detrimental effect on the high-temperature performance of the composite-modified asphalt. This is because bio-oil increases the light components in asphalt and reduces the stiffness of asphalt. Notably, the composite-modified asphalt with a 2% bio-oil content exhibits a more pronounced difference in *Jnr* between the top and bottom sections of the aluminum tube, suggesting that the bio-oil content influences its storage stability to some extent.

**Figure 10:**
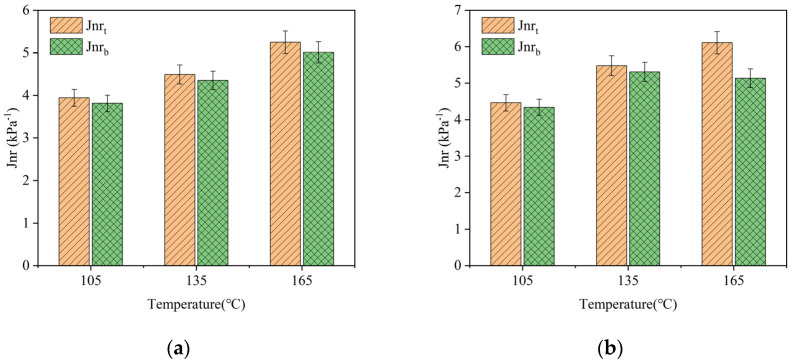
Jnr of composite-modified bitumen at different storage temperatures: (**a**) 0.1 kPa; (**b**) 3.2 kPa.

To investigate the effect of storage temperature on the Jnr of the composite-modified asphalt, Multiple Stress Creep Recovery (MSCR) tests were conducted on the asphalt samples with a 1% bio-oil content, which were stored at different temperatures for 24 h. The results of the *Jnr* are shown in Figure 10.

The bio-oil/lignin composite-modified asphalt has a higher *Jnr* at higher storage temperatures than at lower storage temperatures, as shown in Figure 10. The Jnr of the composite-modified asphalt under the aluminum tube is smaller than that of the composite-modified asphalt on the top part of the aluminum tube. With the increase in storage temperature, the difference becomes larger, indicating that the storage stability of bio-oil/lignin composite-modified asphalt is not good when stored at higher temperatures.

### 3.5. Functional Group Composition Characterization

**Figure 11:**
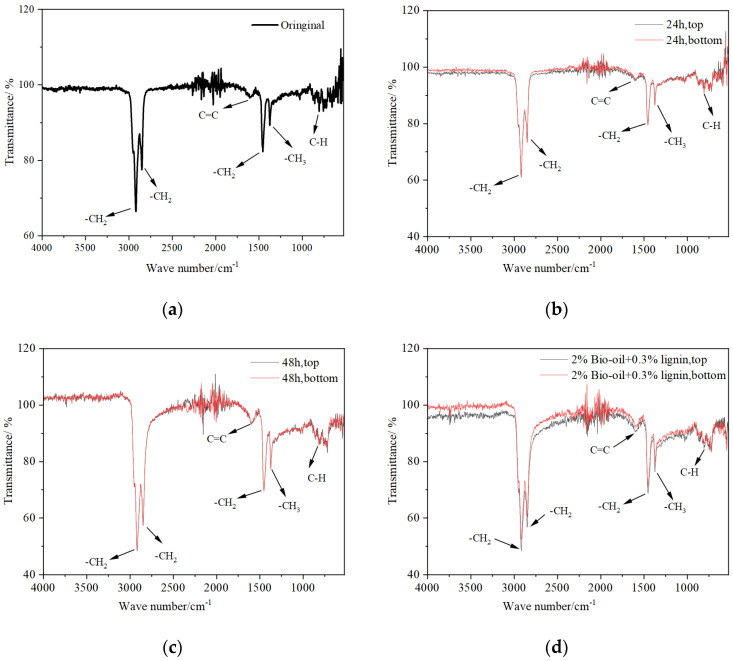

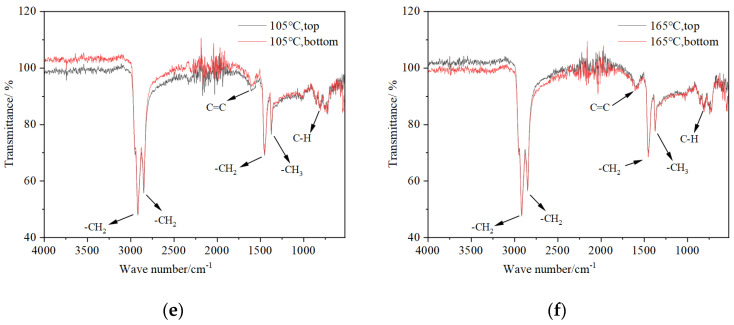
Infrared spectrogram of bio-oil/lignin composite-modified asphalt: (**a**) The 1% bio-oil content is not stored; (**b**) 1% bio-oil content, storage temperature 135 °C, storage for 24 h; (**c**) 1% bio-oil content, storage temperature at 135 °C, storage for 48 h; (**d**) 2% bio-oil content, storage temperature 135 °C, storage for 24 h; (**e**) 1% bio-oil content, storage temperature 105 °C, storage for 24 h; (**f**) 1% bio-oil content, storage temperature 165 °C, storage for 24 h.

To investigate the influence of storage time, temperature, and bio-oil content on the storage stability of composite-modified asphalt, the infrared spectrum of the composite-modified asphalt was obtained by scanning it using a Fourier-transform infrared spectrometer. Figure 11 shows the infrared spectrum of bio-oil/lignin composite-modified bitumen from bio-oil.

Based on the observations depicted in Figure 11, it is evident that the infrared spectra curves and peak values of the asphalt samples, both from the top and bottom sections, exhibit remarkable similarity before and after storage, as well as after different storage times. The six prominent absorption peaks located at 2917 cm^−1^, 2849 cm^−1^, 1605 cm^−1^, 1457 cm^−1^, 1375 cm^−1^, and 808 cm^−1^ consistently appear across the spectra. The peak at 808 cm^−1^, falling within the fingerprint region (1500–600 cm^−1^), primarily arises from the out-of-plane bending vibration of C-H bonds. Its less pronounced intensity suggests the presence of a small amount of aromatic compounds in the composite-modified asphalt. The characteristic peak at 1375 cm^−1^ is attributed to the symmetric bending vibration of C-H bonds on -CH_3_ groups. The peaks near 1457 cm^−1^ are associated with the asymmetric bending vibration of C-H bonds on -CH_2_ groups, indicating the presence of long-chain and branched-chain aliphatic compounds in the composite-modified asphalt. The peak at 1605 cm^−1^, located in the third region (1900–1500 cm^−1^), primarily arises from the breathing vibration of asymmetric benzene rings, indicating the presence of aromatic compounds in the composite-modified asphalt. The peaks at 2917 cm^−1^ and 2849 cm^−1^, falling within the first region (4000–2500 cm^−1^), represent the asymmetric and symmetric absorption vibrations of C-H bonds on methylene (-CH_2_) groups, respectively, indicating the presence of saturated hydrocarbon compounds in the composite-modified asphalt. The overlapping infrared spectra curves and peaks under different bio-oil content and storage temperatures suggest that no new functional groups are formed, indicating that no chemical reactions occur in the bio-oil/lignin composite-modified asphalt during the thermal storage process.

### 3.6. SEM Test

**Figure 12:**
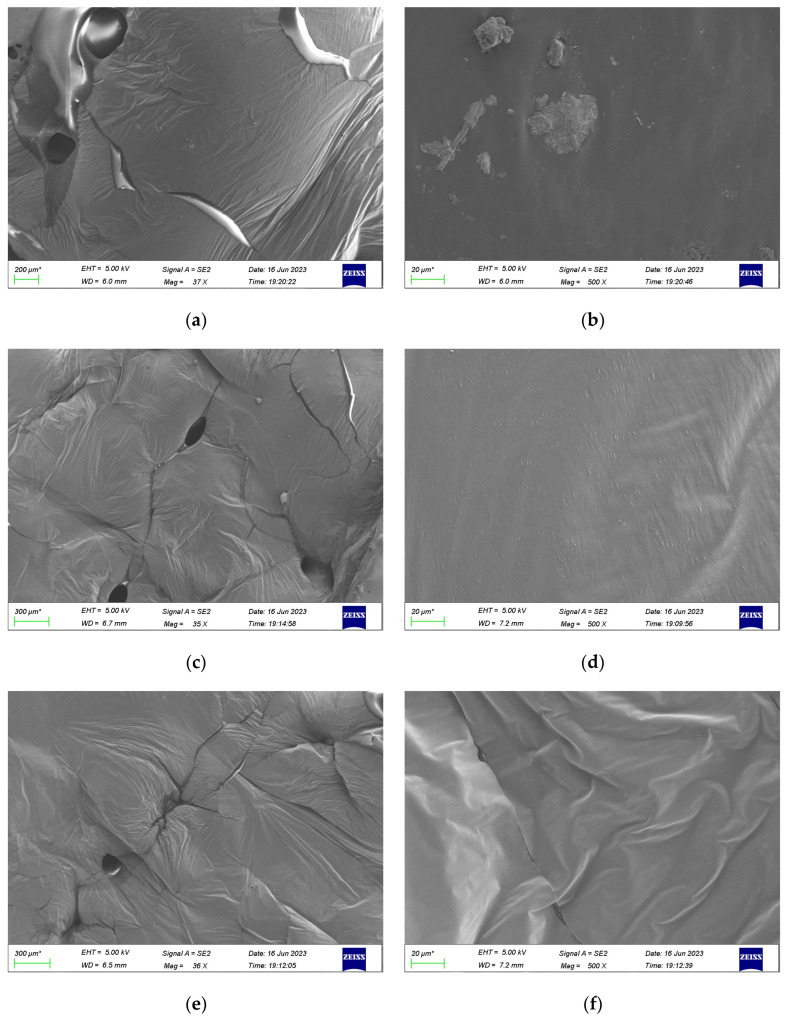

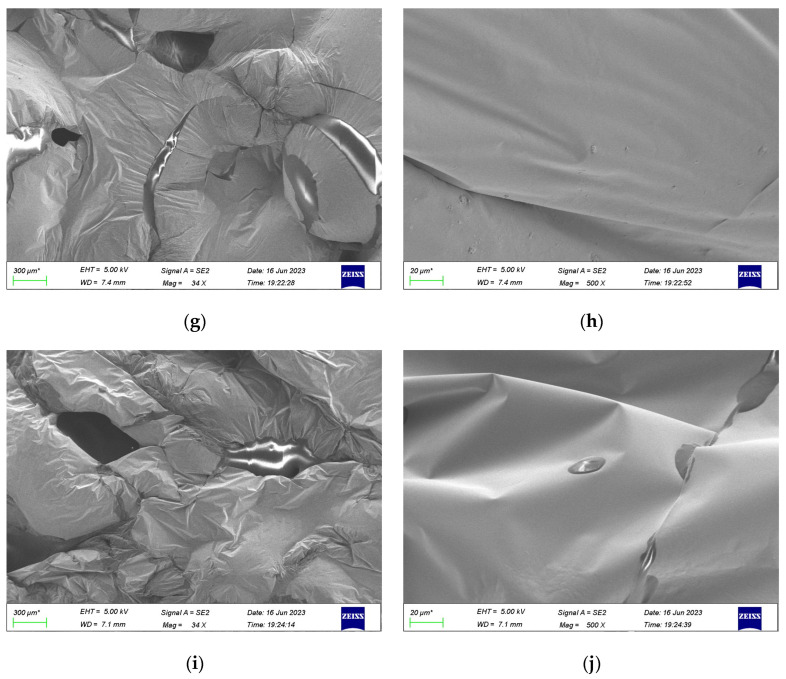
SEM image of bio-oil/lignin composite-modified asphalt: (**a**) original (37×); (**b**): 500×; (**c**) the top part of the aluminum tube specimen was stored for 24 h (35×); (**d**) 500×; (**e**) the bottom part of the aluminum tube specimen was stored for 24 h (36×); (**f**) 500×; (**g**) the top part of the aluminum tube specimen was stored for 48 h (34×); (**h**) 500×; (**i**) the bottom part of the aluminum tube specimen was stored for 24 h (34×); (**j**) 500×.

Scanning electron microscopy (SEM) enables comprehensive observation of the microscopic morphology and structural composition of materials, allowing for the study of asphalt’s nano-level structure [44]. In this experiment, the top and bottom parts of the bio-oil/lignin composite-modified asphalt were observed by scanning electron microscope (SEM) when stored for different times, and whether the stratification and segregation occurred after storage. SEM images of bio-oil/lignin composite-modified bitumen are shown in Figure 12.

From Figure 12a,b, the lignin and asphalt are not miscible and are physically dispersed in asphalt mortar, and the shape of lignin is somewhat irregular, with a block-like and strip-like distribution, which plays the role of filling and viscosity. According to Figure 12c–f, after 24 h of storage, the top asphalt sample contains some lignin particles, while the bottom does not have lignin particles, indicating that after 24 h of storage, the physical stratification phenomenon occurs, and the lignin moves upward, does not maintain structural stability with the bituminous stable network structure, and the bio-oil/lignin composite-modified asphalt at this time is an inhomogeneous system. At the same time, the folds of the lower specimen are more obvious, indicating that the lower specimen has poor fluidity but improves the interfacial viscoelasticity. After 48 h of storage, as shown in Figure 12g–j, only a small part of the lignin in the top asphalt sample moved upward, indicating that the research on composite-modified asphalt tended to be stable at this time, and the folds of the bottom sample were more obvious, which reduced its fluidity.

## 4. Conclusions

In this study, bio-oil and lignin were used as modifiers to modify the matrix asphalt, and the effects of different bio-oil content on the rheological property and storage stability of composite-modified asphalt were discussed. In addition, the effects of different storage temperatures and storage time on the storage stability and high-temperature rheological property of composite-modified asphalt were studied. The main conclusions are as follows:

- Based on the softening point difference, storage stability index Is, segregation rate *Rs*, and *Jnr*, it was found that the composite-modified asphalt had excellent storage stability;
- The high-temperature performance of the composite-modified asphalt after thermal storage has been improved, and the high-temperature performance index of the bottom of the composite-modified asphalt is slightly better than that of the top, which may be caused by the downward migration of heavy components in the asphalt during storage;
- Based on FTIR spectroscopy, it was found that no new functional groups were produced during thermal storage, and no chemical reaction occurred during thermal storage. The results of scanning electron microscopy showed that the longer storage time would make the storage effect of composite-modified asphalt better, which was consistent with the results of softening spread, storage stability index Is, and Rs.

This paper confirms that bio-oil and lignin composite-modified asphalt exhibit good storage stability through multiple indicators, laying a foundation for the practical application of bio-oil and lignin composite-modified asphalt. There are many types of bio-oils, and this study only used a representative wood bio-oil. The future work involves investigating the possibility of increasing the dosage of bio-oil and lignin as much as possible without compromising the performance of the asphalt.

## Figures and Tables

**Table 1:** Properties of base asphalt binder.

Item	Units	Test Results	Standard
Penetration (25 °C, 100 g, 5 s)	0.1 mm	67	JTG-T0604-2011 [35]
Softening temperature	°C	51.0	JTG-T0606-2011 [35]
Ductility (15 °C, 5 cm/min)	cm	>100	JTG-T0605-2011 [35]
RTFO treated at 163 °C, for 85 min			
Quality change	%	0.2	JTG-T0610-1-2011 [35]
Residual penetration ratio (25 °C)	%	62.7	JTG-T0610-2-2011 [35]
Residual ductility (5 °C)	cm	11.2	JTG-T0605-2011 [35]

Note: RTFO—rolling thin film oven.

**Table 2:** Softening point of different asphalts after thermal storage at different temperatures.

Modified Asphalt with Different Bio-Oil Content (%)	Storage Time (h)	Evaluation Index	Softening Point (°C)		
			105	135	165
1	0	-	46.8		
2			44.4		
1	24	TR&B_t_	46.3	47.3	47.5
		TR&B_b_	47.1	46.9	47.4
		ΔTR&B	0.8	0.4	0.1
2		TR&B_t_	43.9	44.5	45.1
		TR&B_b_	44.8	44.8	44.9
		ΔTR&B	0.9	0.3	0.2
1	48	TR&B_t_	47.1	47.6	47.6
		TR&B_b_	47.5	48.0	47.9
		ΔTR&B	0.4	0.4	0.3
2		TR&B_t_	45.2	44.7	45.0
		TR&B_b_	45.4	45.0	44.9
		ΔTR&B	0.2	0.3	0.1

## Data Availability

The original contributions presented in the study are included in the article; further inquiries can be directed to the corresponding author.
